# Sporadic meningioangiomatosis-associated atypical meningioma mimicking parenchymal invasion of brain: a case report and review of the literature

**DOI:** 10.1186/1746-1596-5-39

**Published:** 2010-06-18

**Authors:** Yan-yang Chen, Xiao-ying Tiang, Zhi Li, Bo-ning Luo, Quan Huang

**Affiliations:** 1 Department of Pathology, the First Affiliated Hospital, Sun Yat-sen University. 58 Zhongshan Road II, Guangzhou 510080, China; 2 School of Chinese Medicine, Hong Kong Baptist University. 7 Baptist University Road, Kowloon Tong, Hong Kong, China; 3 Department of Radiology, the First Affiliated Hospital, Sun Yat-sen University. 58 Zhongshan Road II, Guangzhou 510080, China; 4 Department of Neurosurgery, the First Affiliated Hospital, Sun Yat-sen University. 58 Zhongshan Road II, Guangzhou 510080, China

## Abstract

Meningioangiomatosis is a rare hamartomatous lesion or meningiovascular malformation in brain. In extremely rare condition, meningioma may occur together with meningioangiomatosis, and only 19 cases have been described in English literature until now. We now report a case of meningioangiomatosis-associated meningioma with atypical and clear cell variant. A 34-year-old man presented a 3-month history of progressive numbness and weakness of his left lower extremity. He had no stigmata of neurofibromatosis type 2. Magnetic resonance imaging (MRI) revealed multifocal lesions in the right frontoparietal lobe. The lesions were totally removed. Microscopically, parts of lesions were atypical and clear cell meningioma corresponding to WHO grade II. The adjacent brain parenchyma showed the histological features of meningioangiomatosis. Neoplastic cells in atypical meningioma area were immunoreactive to epithelial membrane antigen (EMA) with high MIB-1 index of up to 20%. However, the spindle cells in meningioangiomatosis area were negative for EMA with low MIB-1 index of up to 1%. The diagnosis of atypical meningioma associated with sporadic meningioangiomatosis was made. To our knowledge, this is the first case of a meningioangiomatosis-associated meningioma with atypical and clear cell variant component to be described. The patient had been followed-up for 11 months without adjuvant radiotherapy or chemotherapy. No tumor recurrence was found during this period. Meningioangiomatosis-associated meningioma is more likely to occur in younger patients and histologically to mimic parenchymal invasion of brain. We suggest that postoperative radiotherapy or chemotherapy should be given careful consideration to avoid over-treatment due to erroneously interpret as malignant meningioma.

## Background

Meningioangiomatosis is a rare meningiovascular malformation in the central nervous system, which is characterized by a plaque-like or nodular mass within the cerebral cortex and overlying leptomeninges in the patients with intractable seizures. Meningioangiomatosis may occur either sporadically or occur in patients with neurofibromatosis type 2 (NF2). Sporadic meningioangiomatosis usually presents seizures and persistent headaches. NF2-associated meningioangiomatosis is often asymptomatic and diagnosed only at autopsy [1]. Histologically, the lesion typically presents perivascular proliferation of meningiothelial and fibroblast-like cells, entrapped glial islands and focal calcification. In extremely rare condition, meningioma may occur together with meningioangiomatosis, and only 19 cases have been described in English literature until now [2-14]. Herein, we report a case of meningioma associated with sporadic meningioangiomatosis occurred in a middle-aged man. In contrast to most meningioangiomatosis-associated meningiomas exhibited bland cytological features and corresponding to WHO grade I, our case presented a characteristic of atypical and clear cell meningioma component corresponding to WHO grade II. To our knowledge, this is the first report of meningioangiomatosis-associated meningioma with atypical and clear cell variants.

## Case presentation

**Figure 1:**
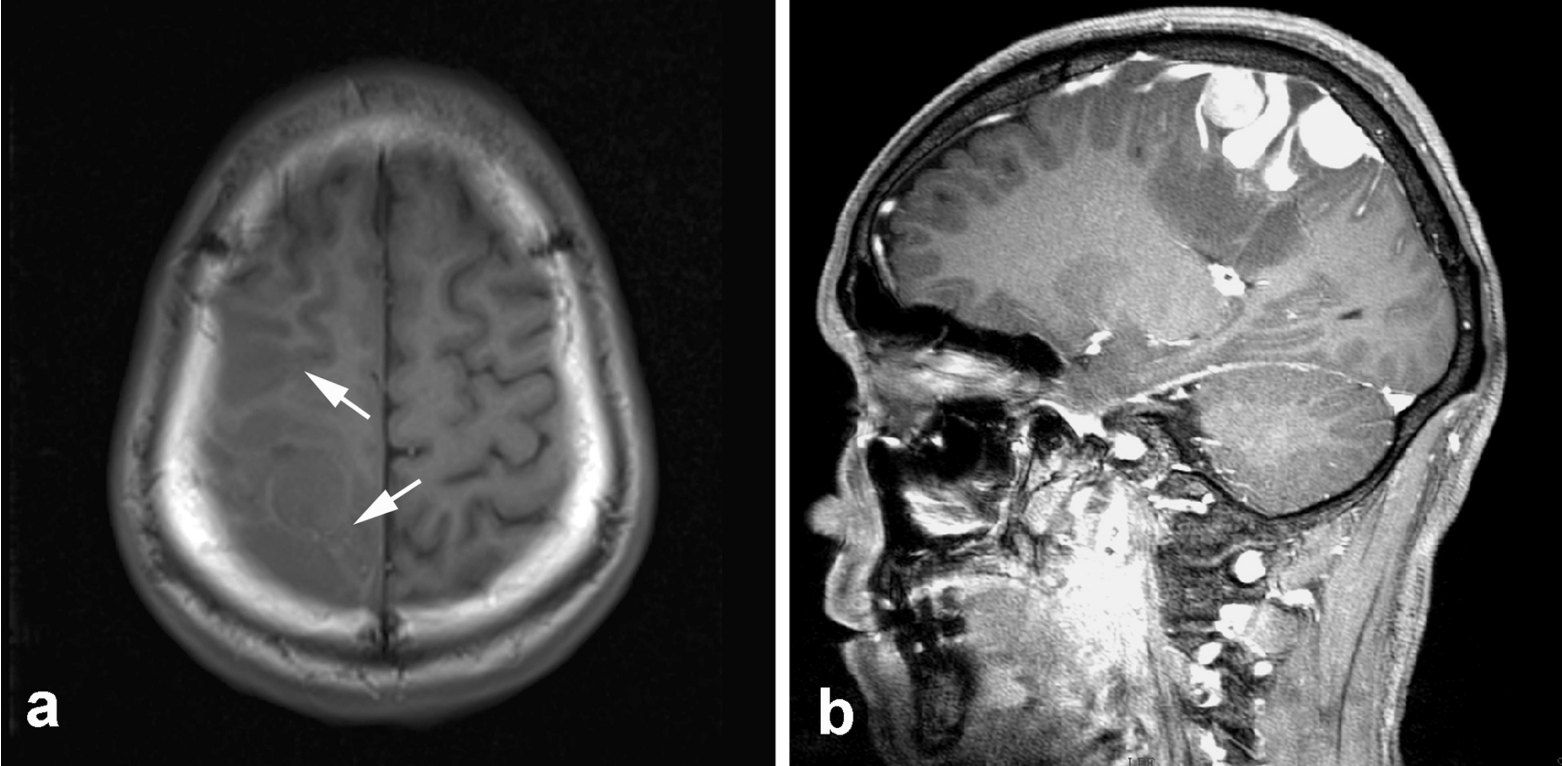
**Neuroimaging findings of lesion. **(a) Preoperative axial T-1 weighted magnetic resonance imaging (MRI) revealed multifocal hypointense lesion localized in right frontoparietal lobe (white arrowed). (b) Sagittal MRI shows the well-circumscribed enhanced lesion extending from the superficial cortex along the meninges into the depth of white matter.

**Figure 2:**
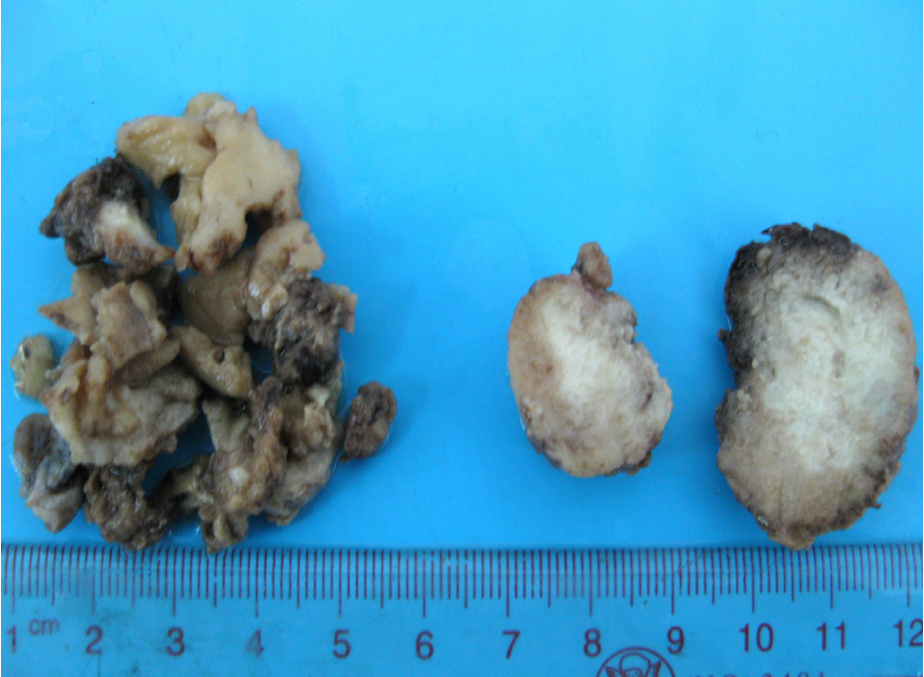
**Gross examination of lesion. **On macroscopical examination, the lesion was composed of grayish smaller tissue fragments of variable sizes and a whitish ovoid mass, measuring 3.0 cm in diameter. No necrosis, haemorrhage and gross calcification were found in the mass and other tissue fragments.

A 34-year-old Chinese man presented a 3-month history of progressive numbness and weakness of his left lower extremity, and which had increased in severity over the past 2 weeks. Epileptic fits or neurological deficits were not recorded. On physical examination, the patient had mild weakness on his left lower extremity. Sensory examination revealed that hypoalgesia could be detected in the area below the left knee. Neurological examinations showed no abnormality in cranial nerves, and no defect was disclosed by the visual field test. The patient had no family history of neurofibromatosis, and no stigmata of NF2 including acoustic schwannomas or masses of the spinal cord was found by neuroimaging examination. Magnetic resonance imaging (MRI) revealed multiple hypointense lesions in the right frontoparietal lobe with heterogeneous contrast enhancement after Gd-diethylenetriamine pentaacetic acid administration (Figure 1). Fluid attenuated inversion recovery (FLAIR) demonstrated predominantly gyriform hyperintensity and leptomeinges. The patient underwent a right frontal craniotomy. A well-demarcated ovoid mass with multiple yellow firm granulations scattered on the cortical surface was totally removed. On macroscopical examination, part of lesion tissue appeared grayish and moderately firm with smaller tissue fragments of variable sizes. The whitish and slightly hard ovoid mass, measuring 3.0 cm in diameter, was well-demarcated without fibrous capsule and gross calcification. Neither necrosis nor haemorrhage was found in the mass and other tissue fragments (Figure 2).

**Figure 3:**
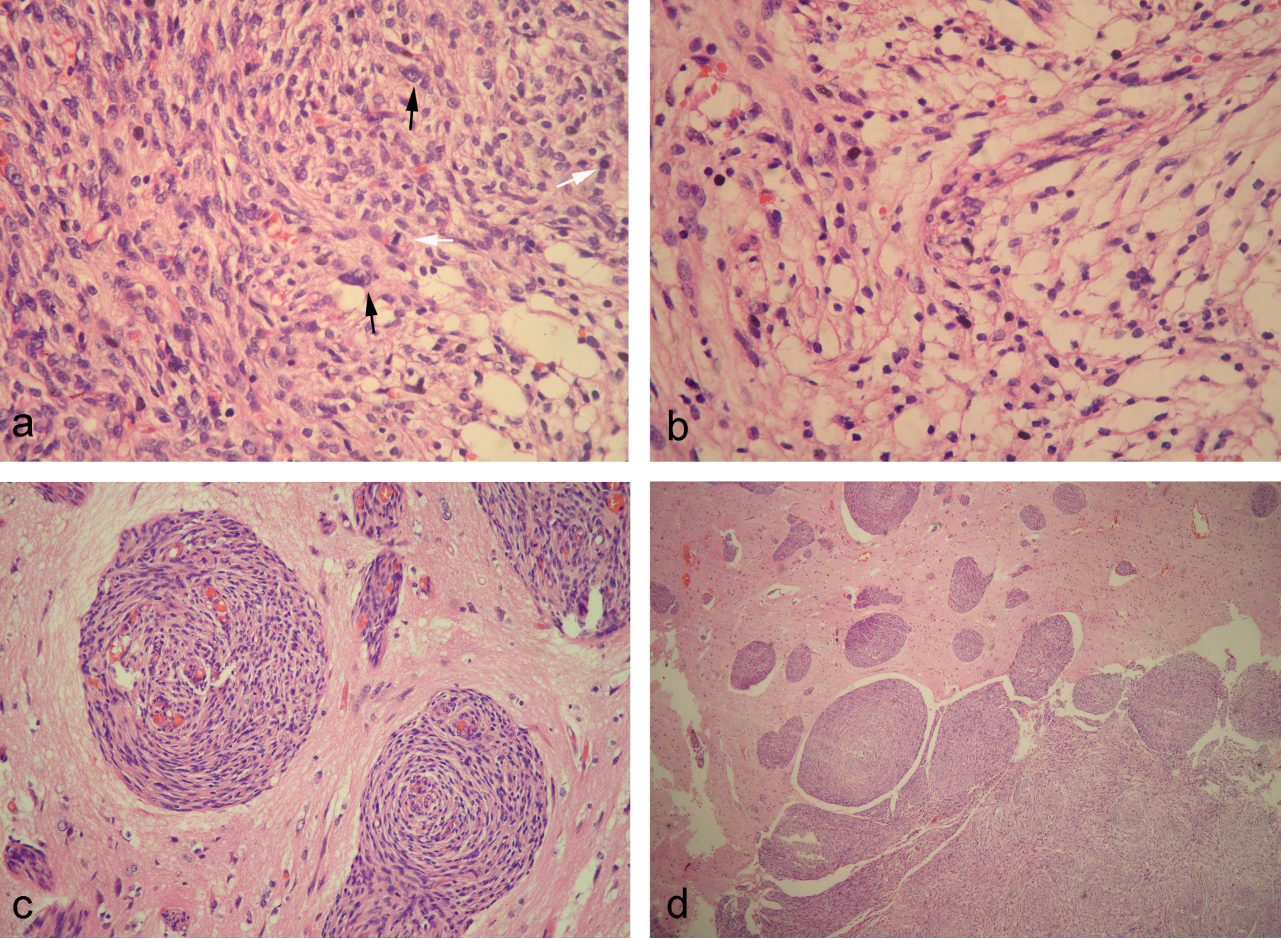
**Histopathological findings of lesion. ** (a) Photomicrographs showed that focal hypercellularity and atypia were noted in whitish mass with higher mitotic activity (white arrowed) and enlarged polymorphic nuclei (black arrowed). (b) In some area, the mass was composed of mainly clear cells with small round monomorphic nuclei and abundant clear cytoplasm resembling the histological feature of clear cell variant of meningioma. (c) The adjacent brain parenchyma to the mass and other removed smaller grayish lesion revealed numerous abnormal small intracortical blood vessels, which were cuffed by meningothelial or fibroblasts-like spindle cells with bland nuclei and elongated cytoplasm. (d) The gradual transition from perivascular proliferation of spindle cells to the mass of the meningioma could be observed. (a & b, H&E staining with original magnification of 400×; c, H&E staining with original magnification of 200×; d, H&E staining with original magnification of 100×).

**Figure 4:**
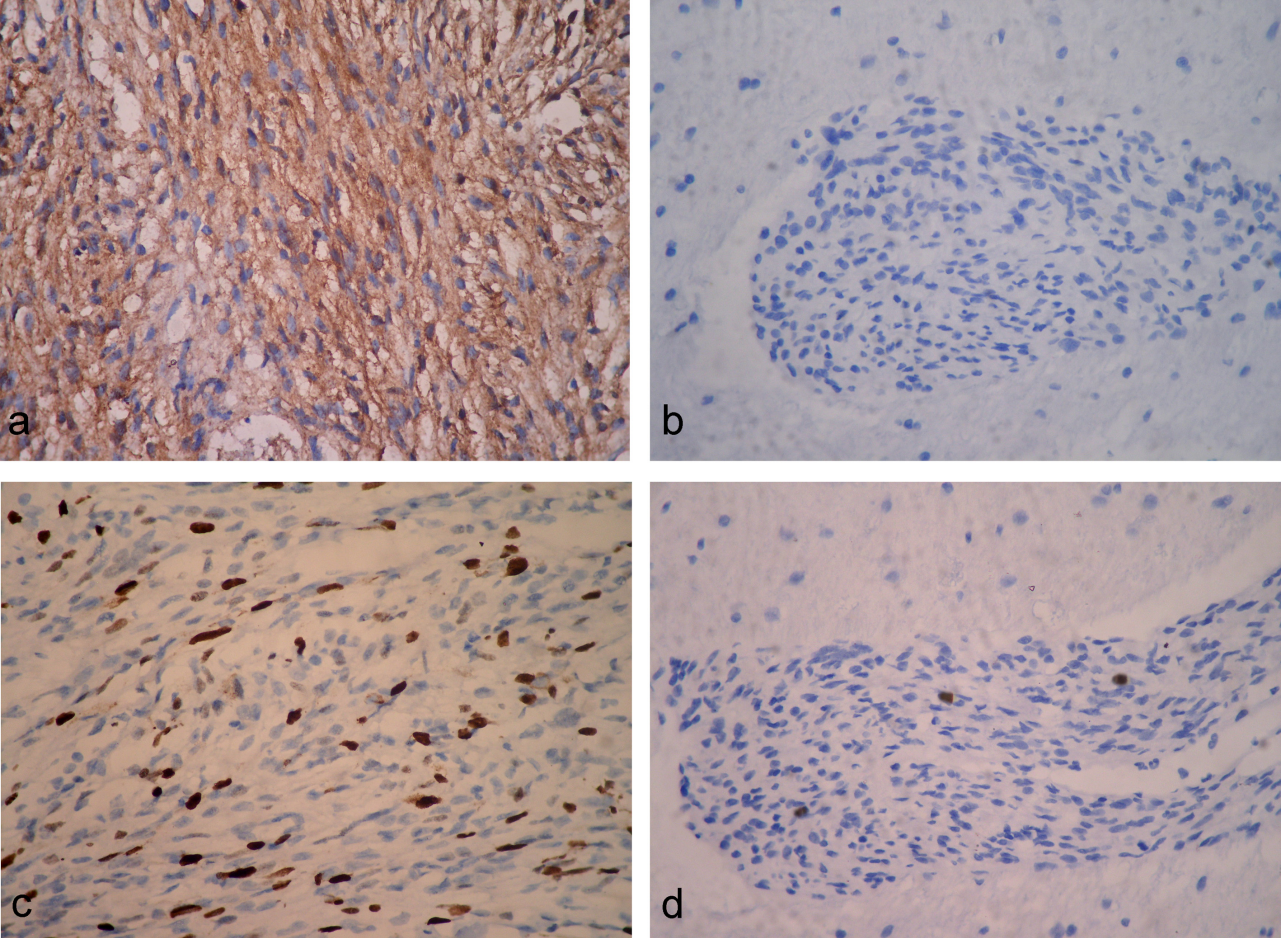
**Immunohistochemical staining of lesion. **Immunohistochemical staining for EMA demostrated the neoplastic cells were focally positive in atypical meningioma area (a), but lack of immunoreactivity in the spindle cells of meningioangiomatosis area (b). Higher cellular proliferation was observed in the atypical meningioma area with approximately 20% of MIB-1 index (c), but lower MIB-1 index of up to 1% was detected in the spindle cells of meningioangiomatosis area (d). (a-d, immunohistochemical staining with original magnification of 400×)

It was found the removed whitish mass was composed of collagen bundles and spindle tumor cells forming small whorls and syncytial structures when examined under the light microscope. Focal hypercellularity and higher mitotic activity were noted. Necrosis and haemorrhage were not observed, but up to 6 mitotic figures of tumor cells per 10 high power fields were detected in these areas (Figure 3a). These histopathological findings were consistent with atypical meningioma. In some areas, the mass was mainly composed of clear cells resembling the classical histological feature of clear cell meningioma with small round monomorphic nuclei and abundant clear cytoplasm. Neither mitosis nor necrotic areas were seen (Figure 3b). The adjacent brain parenchyma to the mass and other removed smaller grayish lesion revealed numerous abnormal small intracortical blood vessels, which were cuffed by meningothelial or fibroblast-like spindle cells (Figure 3c). The spindle cells showed bland nuclei and elongated cytoplasm with indistinct cell borders. Neither necrosis nor mitotic activity was found. Gliosis was observed in the intervening brain parenchyma. The gradual transition from perivascular proliferation of spindle cells to the mass of the meningioma could be observed (Figure 3d). Immunohistochemically, the solid mass was positive to epithelial membrane antigen (EMA) and vimentin, but the perivascular proliferating spindle cells showed no immunoreactive for EMA (Figure 4a and 4b). Cytokeratin (CK), glial fibrillary acidic protein (GFAP), CD34, Desmin, CD99 and S-100 were negative in both components of lesion. MIB-1 index was high with focally 20% in solid mass of lesion. However, there was only up to 1% of MIB-1 index in perivascular spindle cells (Figure 4c and 4d). Based on these findings, a histological diagnosis of atypical meningioma (WHO grade II) associated with meningioangiomatosis was made.

During postoperative course, the patient developed a transient mild left hemiparesis within 2 weeks after surgery. When he was recovered from this condition, the patient reported an improvement of his sensorimotor problems, but slight numbness remained in his left lower extremity. The patient was discharged home without adjuvant radiotherapy or chemotherapy, and 11-month follow-up was performed till the patient was lost to be contacted. Follow-up MRI did not show any residual lesion 3 months after surgery, and neither tumor recurrence nor seizure was found during this period of postoperative follow-up.

Meningioangiomatosis is a rare malformative or hamartomatous lesion involved in meninges and cortex. It was first described in 1915 by Bassoe and Nuzum as an incidental autopsy finding in a boy with NF-2 [15]. In 1937, Worster-Drought et al reported a similar case and named it as "meningioangiomatosis" because the researchers considered it was a "forme fruste" of NF [16]. Although meningioangiomatosis was originally described in association with NF-2, the recent study by Omeis et al [17] has been revealed that meningioangiomatosis occurred more frequently in sporadic form and only 14 cases (14%) of the 100 reported cases are associated with NF. Similarly, Takeshima et al also observed this association in only 25% of patients with meningioangiomatosis, whereas the other 75% patients occurred only sporadically [18].

The pathogenesis of meningioangiomatosis remains unclear, it has been debated whether the biological nature is a hamartoma or a neoplasm. For a long time, meningioangiomatosis was considered as a harmartomatous lesion with degenerative change [15]. Other main hypotheses of meningioangiomatosis include cortical vascular malformation [19,20] and direct invasion of the brain parenchyma by a leptomeningeal-based meningioma [4]. Some studies revealed that loss of 22q12 (NF2 gene) in an each case of meningioangiomatosis and meningioangiomatosis-associated meningioma indicated the possibility of which meningioangiomatosis may be neoplastic in nature [8,19]. However, recent large genetic series demonstrated that there was no NF2 gene mutation either in sporadic or NF2-associated meningioangiomatosis. These findings support the hypothesis of hamartomatous nature of meningioangiomatosis [21,22]. In our case, we found the MIB-1 index was consistently higher in the meningioma component rather than meningioangiomatosis component, and there were lack of EMA-positive cells in the proliferative perivascular areas in meningioangiomatosis component. These observations suggest that meningioangiomatosis had distinct histological and biological features to meningioma. Regardless its etiology, we consider meningioangiomatosis should be best categorized as a hamartomatous or malformative lesion rather than a neoplasm in nature.

Meningioangiomatosis has rarely been reported to coexist with meningiomas. To our knowledge, only 19 cases of meningioma together with meningioangiomatosis have been reported in English literature to date [2-14]. Among these patients, there was an obvious male tendency of male to female ratio being approximately 3:1. It contrasts to usual prevalence of meningioma in female population (2:1 female/male ratio in intracranial meningiomas and 4:1 in spinal meningiomas). Meningioangiomatosis-associated meningiomas seem to occur frequently in young patients with the mean age of 16.0 years (ranging from 9 months to 58 years old), although other conventional meningiomas occur most commonly in middle-aged or elderly patients. None of these patients was associated with NF-2. The most common symptom we observed in the literature review was seizure. Histologically, most of meningiomas were frequent meningioma variants, including 9 transitional meningiomas, 5 fibroblastic meningiomas, and 1 meningothelial meningioma. There were only three rare variants recorded in previous literatures: 1 atypical meningioma, 1 microcystic meningioma and 1 "sclerosing variant". The last one was not a widely known histological variant of meningioma characterized by extensive collagen deposition with focal cellular nests of meningothelial cells (Table 1). Our case described a meningioangiomatosis-associated meningioma occurred in a middle-aged man with atypical variant component and clear cell variant component, both of which correspond to WHO grade II. To our knowledge, this is the first case of a meningioangiomatosis-associated meningioma with clear cell variant component. Like atypical meningioma, clear cell meningioma is also an uncommon variant of meningioma. Mixed meningiomas combined with different variant of meningioma, including mixed atypical and clear cell meningioma, have rarely been reported. However, they are firstly found in meningioangiomatosis-associated meningioma in our case. The findings in this case indicate that meningioangiomatosis-associated meningioma might be composed of any variants of meningioma either in sole pattern or composite pattern.

**Table 1:** Clinicopathological features of meningioangiomatosis associated with meningiomas observed in present and previous reports

*Authors*	*Case*	*Age (yr)/sex*	*site*	*Clinical presentation*	*NF-2*	*Type of meningioma*	*Outcome*
Auer RN (1982) [2]	1	15/male	Frontal lobe	Subarachnoid haemorrhage	No	Fibroblastic	Dead due to operative complication
Louw D (1990) [3]	2	15/male	Frontal lobe	Subarachnoid haemorrhage	No	Fibroblastic	Not recorded
		33/male	Frontal lobe	Headache	No	Transitional	Not recorded
Wilson D (1991) [4]	1	17/male	Frontal lobe	Headache and seizure	No	Transitional	Not recorded
Blumenthal D (1993) [5]	1	11 mo/male	Frontal lobe	Seizure	No	Transitional	No recurrence
Giangaspero F (1999)[6]	2	9/male	Temporal lobe	Asymptomatic	No	Transitional	No recurrence
		28/male	Frontal lobe	Seizure	No	Transitional	No recurrence/alive
Mut M (2000) [7]	1	20/female	Temporal lobe	Seizure	No	Transitional	No recurrence/alive
Sinkre P (2001) [8]	1	8/male	Frontal lobe	Headache	No	Atypical	No recurrence/alive
Kim NR (2002) [9]	5	3/male	Frontoparietal	Seizure	No	Fibroblastic	No recurrence/alive
		4/male	Frontal lobe	Sudden headache	No	Fibroblastic	No recurrence/alive
		6/male	Temporal lobe	Headache and facial pasly	No	Transitional	Recurrence/alive
		9/male	Frontal lobe	Seizure	No	Meningothelial	No recurrence/alive
		19/male	Temporal lobe	Seizure	No	Sclerosing	No recurrence/alive
Meyer S (2002) [10]	1	4/female	Temporal lobe	Seizure	No	Fibroblastic	No recurrence
Iezza G (2003) [11]	1	33/male	Frontal lobe	Seizure	No	Transitional	No recorded
Kuchelmeister K (2003) [12]	1	58/male	Frontal lobe	Headache and forgetfulness	No	Microcystic	No recurrence
Deb P (2006) [13]	1	1.5/female	Temporal lobe	Seizure	No	Transitional	No recurrence
Saad A (2009) [14]	1	3/female	Frontal lobe	Seizure	No	-	No recorded
Present study	1	34/male	Frontoparietal	Weakness of low extremity	No	Atypical and clear cell variant	No recurrence/alive

The main differential diagnosis of meningioangiomatosis-associated meningioma to be considered on histopathology is malignant meningioma with invasion of brain parenchyma because infiltration of cortex by the perivascular proliferating spindle cells and reactive gliosis sometimes may be erroneously interpreted as invasion of brain. However, true parenchymal invasion is present when neoplastic cells break through the pia mater to involve the underlying cortex and encircle islands of heavily gliotic tissue. In our case, meningioma components of the lesion mixed atypical cells and clear cells with hypercellularity, higher mitotic activity and higher MIB-1 index which corresponded to WHO grade II; however, the fibroblast-like spindle cells disposed around the blood vessels were bland appearance with lower proliferation index and lacking EMA expression. Therefore, we did not regard the meningioangiomatosis component of the lesion as the brain invasion by atypical or clear cell meningioma because high level of MIB-1 index is known to be detected in meningiomas of brain invasion [23]. These findings suggest encircled gliotic cortical tissue is not true invasion but a condition simulated by meningioangiomatosis. Cases of meningioangiomatosis with meningioma usually had a favourable outcome after surgical resection. In previously reported cases, only one case was dead due to operative complication [2], and another one had recurrence after surgery [9]. In our case, a follow-up period of 11 months was performed but the patient did not show any sign of tumor recurrence or seizure. In contrast to the pervious cases with conventional meningioma variants, ours presented a more aggressive meningioma variant corresponding to WHO grade II. Further investigation with more cases and longer follow-up data need to be performed to clarify whether or not aggressive variant of meningioma might result in a worse prognosis of meningioangiomatosis-associated meningioma.

## Conclusion

We reported a rare case of meningioangiomatosis-associated meningioma with atypical and clear cell meningioma variants occurring in frontoparietal lobe of a middle-aged man. In contrast to conventional meningiomas, meningioangiomatosis-associated meningioma is more likely to occur in younger males, and any variants of meningioma might present in this lesion with either sole pattern or composite pattern. Although meningioangiomatosis is a benign hamartomatous or malformative lesion, it may be erroneously interpreted as brain invasion when it uncommonly co-existed with meningioma. The relationship between the prognosis and meningioangiomatosis-associated aggressive meningioma remains unclear. The investigations of a longer follow-up period and more cases would help to better clarify the biological characteristics and clinical outcome of this rare but distinct lesion.

## Competing interests

The authors declare that they have no competing interests.

## Authors' contributions

YYC made contributions to acquisition of clinical data, and analysis the histological features of case by H&E staining. TXY drafted the manuscript. ZL revised manuscript critically for important intellectual content and has given final approval of the version to be published. BNL participated in the design of the study and performed the neurological image analysis. QH carried out the immunoassays. All authors read and approved the final manuscript.

## Consent

Written informed consent was obtained from the patient for publication of this case report and any accompanying images. A copy of the written consent is available for review by the Editor-in-Chief of this journal.
